# Bone density and pain response following intensity-modulated radiotherapy versus three-dimensional conformal radiotherapy for vertebral metastases - secondary results of a randomized trial

**DOI:** 10.1186/s13014-018-1161-4

**Published:** 2018-10-30

**Authors:** Tanja Sprave, Vivek Verma, Robert Förster, Ingmar Schlampp, Katharina Hees, Thomas Bruckner, Tilman Bostel, Rami Ateyah El Shafie, Thomas Welzel, Nils Henrik Nicolay, Jürgen Debus, Harald Rief

**Affiliations:** 1 University Hospital of Heidelberg, Department of Radiation Oncology, Im Neuenheimer Feld 400, 69120 Heidelberg, Germany; 2 Department of Radiation Oncology, Allegheny General Hospital, Pittsburgh, PA USA; 3 Heidelberg Institute of Radiation Oncology (HIRO), Im Neuenheimer Feld 280, 69120 Heidelberg, Germany; 4 University Hospital Zurich, Department of Radiation Oncology, Raemistrasse 100, 8091 Zurich, Switzerland; 5 University Hospital of Heidelberg, Department of Medical Biometry, Im Neuenheimer Feld 305, 69120 Heidelberg, Germany; 6 University Hospital of Freiburg, Department of Radiation Oncology, Robert-Koch-Strasse 3, 79106 Freiburg, Germany

**Keywords:** Bone metastases, Spine, Intensity-modulated radiation therapy, Bone density, Palliative radiotherapy

## Abstract

**Background:**

This was a prespecified secondary analysis of a randomized trial that analyzed bone density and pain response following fractionated intensity-modulated radiotherapy (IMRT) versus three-dimensional conformal radiotherapy (3DCRT) for palliative management of spinal metastases.

**Methods/materials:**

Sixty patients were enrolled in the single-institutional randomized exploratory trial, randomly assigned to receive IMRT or 3DCRT (30 Gy in 10 fractions). Along with pain response (measured by the Visual Analog Scale (VAS) and Chow criteria), quantitative bone density was evaluated at baseline, 3, and 6 months in both irradiated and unirradiated spinal bodies, along with rates of pathologic fractures and vertebral compression fractures.

**Results:**

Relative to baseline, bone density increased at 3 and 6 months following IMRT by a median of 24.8% and 33.8%, respectively (*p* < 0.01 and *p* = 0.048). These figures in the 3DCRT cohort were 18.5% and 48.4%, respectively (p < 0.01 for both). There were no statistical differences in bone density between IMRT and 3DCRT at 3 (*p* = 0.723) or 6 months (*p* = 0.341). Subgroup analysis of osteolytic and osteoblastic metastases showed no differences between groups; however, mixed metastases showed an increase in bone density over baseline in the IMRT (but not 3DCRT) arm. The 3-month rate of the pathological fractures was 15.0% in the IMRT arm vs. 10.5% in the 3DCRT arm. There were no differences in pathological fractures at 3 (*p* = 0.676) and 6 (*p* = 1.000) months. The IMRT arm showed improved VAS scores at 3 (*p* = 0.037) but not 6 months (*p* = 0.430). Using Chow criteria, pain response was similar at both 3 (*p* = 0.395) and 6 (*p* = 0.732) months.

**Conclusions:**

This the first prospective investigation evaluating the impact of IMRT vs. 3DCRT on bone density. Along with pain response and pathologic fracture rates, significant rises in bone density after 3 and 6 months were similar in both cohorts. Future randomized investigations with larger sample sizes are recommended.

**Trial registration:**

NCT, NCT02832830. Registered 14 July 2016

## Introduction

Spinal metastases, which occur in up to 40% of advanced-stage cancer patients, can be a major source of symptomatic burden and quality of life detriment [1]. These include not only pain and immobility, but also neurological deficits and risk of pathological fractures. Historically, conventionally fractionated three-dimensional conformal radiotherapy (3DCRT) has been the technique of choice to palliate these cases [2, 3]. However, the advancement of technologies such as intensity-modulated radiation therapy (IMRT) allows for safer dose-escalation by means of higher conformality, image guidance, and decreased doses to nearby organs-at-risk (OARs).

Because much of current research on spinal metastases involves stereotactic radiotherapy (achieved in 5 or fewer fractions), fractionated IMRT has remained an under-studied option for these cases. Although stereotactic radiotherapy may be accomplished by inverse-planned IMRT, volumetric modulated arc therapy (VMAT), or TomoTherapy techniques, fractionated IMRT (most commonly 30 Gy in 10 fractions as in this study), which can also be performed with any of the aforementioned techniques, has largely been overshadowed to date and thus deserves further study.

There are known serious adverse events associated with spinal irradiation, such as decreases in bone density potentially resulting in vertebral compression fractures (VCFs). There are no randomized data evaluating these parameters in IMRT versus 3DCRT to date. This was a prespecified secondary analysis of a randomized trial, which evaluated bone density and pain response following IMRT versus conventional 3DCRT as part of palliative management of painful spinal metastases.

## Materials and methods

### Trial design and participants

The randomized trial, registered on clinicaltrials.gov (NCT02832830), was approved by the Heidelberg University Independent Ethics Committee (Nr. S-238/2016). Details of the study design have been published previously [4]. The primary endpoint of this randomized, single-institutional, pilot trial was 3-month RT-induced toxicity following delivery of 30 Gy in 10 fractions of image-guided IMRT versus conventional 3DCRT in patients with previously untreated spinal metastases. All patients had an established indication for RT, including pain and/or neurological deficits. The present study was a prespecified secondary analysis of bone density, as well as pain response and rates of pathologic fracture and VCF.

A block randomization approach (block size of 6) was used to ensure that the two groups were balanced. In addition to the above, inclusion criteria were ages 18–85, a Karnofsky performance score ≥ 50, and ability to provide written informed consent. Exclusion criteria were subjects with significant neurological or psychiatric disorders precluding informed consent, previous RT to the given irradiation site, or multiple myeloma or lymphoma histology. Number or location of metastases were not specific criteria for inclusion or exclusion, nor was the presence of spinal cord compression.

### Assessment of endpoints

Per protocol, bone density in irradiated and non-irradiated vertebral bodies, other pathologic vertebral fractures, and VCFs were assessed at baseline and at 3 and 6 months after RT. Bone density was assessed with the Syngo Osteo CT workstation in manually selected regions of interest. Hounsfield units (HU) were used for bone density measurements. The Siemens Somatom Sensation Open (Siemens, Erlangen, Germany) scanner was used for all CT examinations. Measurements were carried out at the appropriate site by a single physician in light of inter-observer bias. During the observation period, because most participants received anti-osteoresoptive treatment, changes in bone density in unaffected vertebrae were also measured.

Pathologic fractures were diagnosed by experienced radiologists by means of CT and/or MRI imaging by comparing to baseline imaging tests. New fractures were, by definition, not present on initial imaging, whereas progressive fractures referred to visibly increasing size and/or number of fracture gaps, dislocation of fracture fragments, or increasing sintering of the VCF. A VCF was defined as the reduction of the vertebral body height by more than 20%. Each of these was grouped under the term of “pathologic fractures”.

In addition to evaluating neuropathic pain, overall pain response to RT was quantified by the visual analog scale (VAS), measured at the irradiated region prior to, immediately following, and at 3 and 6 months after RT. Pain response was designated as complete response (CR), partial response (PR), pain progression (PP), and intermediate pain (IP) according to the International Bone Consensus response categories by Chow et al. [5]. Complete response (CR) was defined as no pain (VAS = 0) after 3 months and partial response (PR) as an improvement by at least two points after 3 and 6 months. CR referred to VAS = 0 with no concurrent increase in analgesic intake (stable or reducing analgesics in daily oral morphine equivalents). PR was pain reduction of 2 or more without increase in analgesics, or analgesic reduction of at least 25% from baseline without an increase in pain. PP was defined as increase in pain score of ≥2 above baseline with stable oral morphine equivalents, or an increase of 25% or more in the latter with the pain score stable or 1 point above baseline. Any response not covered by the CR, PR or PP definitions was called “stable pain”. Responders were defined as having CR or PR, and non-responders as having PP or IP.

Exploratory analysis of overall survival (OS) was performed and defined as the time from initial diagnosis until death or censored at last contact.

### Radiotherapy

CT simulation was performed with custom immobilization using Aquaplast® (Aquaplast Corporation, Wyckoff, NJ, USA) head masks for cervical spine cases and Wingstep/Prostep® (Elekta, Stockholm, Sweden) devices for thoracolumbar cases. In addition to OARs (dose constraints for which were per QUANTEC recommendations), the clinical target volume (CTV) was delineated on the planning CT and encompassed the affected vertebral body [6]. The planning target volume (PTV) was an isotropic 1 cm expansion of the CTV and was to be covered by the 90% isodose line. The prescription dose for both cohorts was 30 Gy in 10 fractions.

The IMRT group received image-guided (mega- or kilo-voltage cone beam computed tomography) RT by means of step-and-shoot IMRT, VMAT (Elekta Versa HD accelerator), or helical TomoTherapy (Accuray Inc., Madison, WI). The 3DCRT cohort was most commonly delivered with two or three anteroposterior 6 MV individually-formed beams. Position verification was applied by weekly kilo-voltage CT and before each fraction by comparing orthogonal portal images with digitally reconstructed radiographs from the planning CT.

### Statistical analysis

Complete details regarding statistical analysis are presented elsewhere [4]. Owing to the exploratory nature of this study, a complete power calculation was not possible; however, with 30 patients in each group, it was possible to detect a standardized mean-value effect of 0.8 with 80% power at a significance level of 0.05.

All variables were analyzed descriptively by tabulation of the measures of the empirical distributions. According to the scale level of the variables, means (Hodges-Lehmann estimates) and standard deviations or absolute and relative frequencies, respectively, were reported. Additionally, for variables with longitudinal measurements, the time courses of individual patients and summarized by treatment groups. Descriptive *p*-values of the corresponding statistical tests comparing the treatment groups were given. Analysis of covariance (ANOVA) with repeated measurements, with treatment group as a factor, and pain medication as covariates, were done. The Wilcoxon rank-sum test was used to detect possible differences between groups after 3 and 6 months. All statistical analyses were done using SAS software Version 9.4 or higher (SAS Institute, Cary, NC, USA).

### Funding source

The sponsors of the study had no role in study design, data analysis, data interpretation and wording of the report. The corresponding author (HR) had full access to the entire data of the study and had final responsibility regarding the decision to submit for publication.

## Results

From November 2016 to May 2017, 60 patients were randomized. No patients were excluded post randomization. Baseline characteristics were balanced between the two treatment arms (Table 1, as previously reported) [7].

**Table 1:** Baseline characteristics of randomly assigned participants

	IMRT group *n* = 30		3D group *n* = 30		*p*-value
	*n*	%	*n*	%	
Age (years)					0.219
Mean (SD)	66,1 (10,5)		62,5 (11,8)		
Karnofsky-Perfomance Status					0.283
Mean (SD)	64,9 (9,32)		61,3 (9,7)		
Gender					0.795
Male	17	56,7	16	53,3	
Female	13	43,3	14	46,7	
Weight (kg, SD)	75,8 (14,9)		76,2 (19,4)		0.929
Height (cm, SD)	171,6 (8,8)		172,2 (8,6)		0.790
Body mass index (BMI)					0.960
Mean (SD)	25,7 (4,4)		25,6 (5,7)		
Primary site					
Lung cancer	11	36,7	16	53,3	
ABreast cancer	7	23,3	6	20	
Prostata cancer	6	20	1	3,3	
Other	6	20	7	23,3	
Volume of metastases at baseline					
Mean size (mm^3^)	30	1166,6	30		0.191
Localization metastases					0.261
Cervical	4	13,3	5	16,7	
Thoracic	15	50	15	50	
Lumbar	11	36,7	7	23,3	
Sacrum	0	0	3	10	
Number metastases					0.140
1 metastase	17	56,7	10	33,3	
2 metastases	4	13,3	9	30	
3 metastases	9	30	11	36,7	
Distant metastases at baseline					
Viszeral	14	46,7	10	33,3	0.292
Lung	7	23,3	6	20	0.754
Brain	4	13,3	5	16,7	0.718
Tissue	5	16,7	5	16,7	1.000
Hormontherapy	12	40	6	20	0.091
Immuntherapy	4	13,3	5	16,7	0.718
Chemotherapy	14	46,7	20	66,7	0.118
Surgery	18	60	13	43,3	0.196
Neurological deficit at baseline	4	13,3	3	10	0.688
Bisphosphonate at baseline	13	43,3	7	23,3	0.100
Orthopedic corset at baseline	9	30	10	33,3	0.781
Medication at baseline					
Sleeping medication	5	16,7	2	6,7	0.228
Psychiatric medication	9	30	6	20	0.371
Opiate	20	66,7	17	56,7	0.426
NSAID	23	76,7	19	63,3	0.260

Explanation: Others: carcinoma of unknown primary (CUP), gastrointestinal stromal tumor (GIST), melanoma, mesothelioma, pancreatic cancer, renal cancer. Abbreviations: *NSAID* nonsteroidal inflammatory drug

**Fig. 1:**
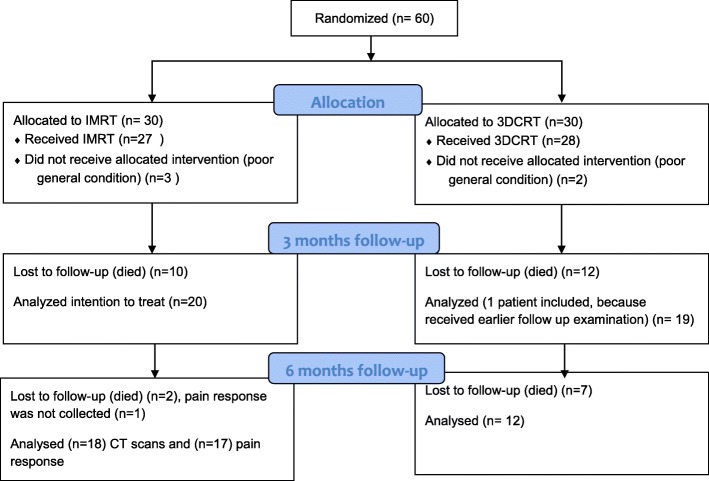
Consort diagram of the trial

**Fig. 2:**
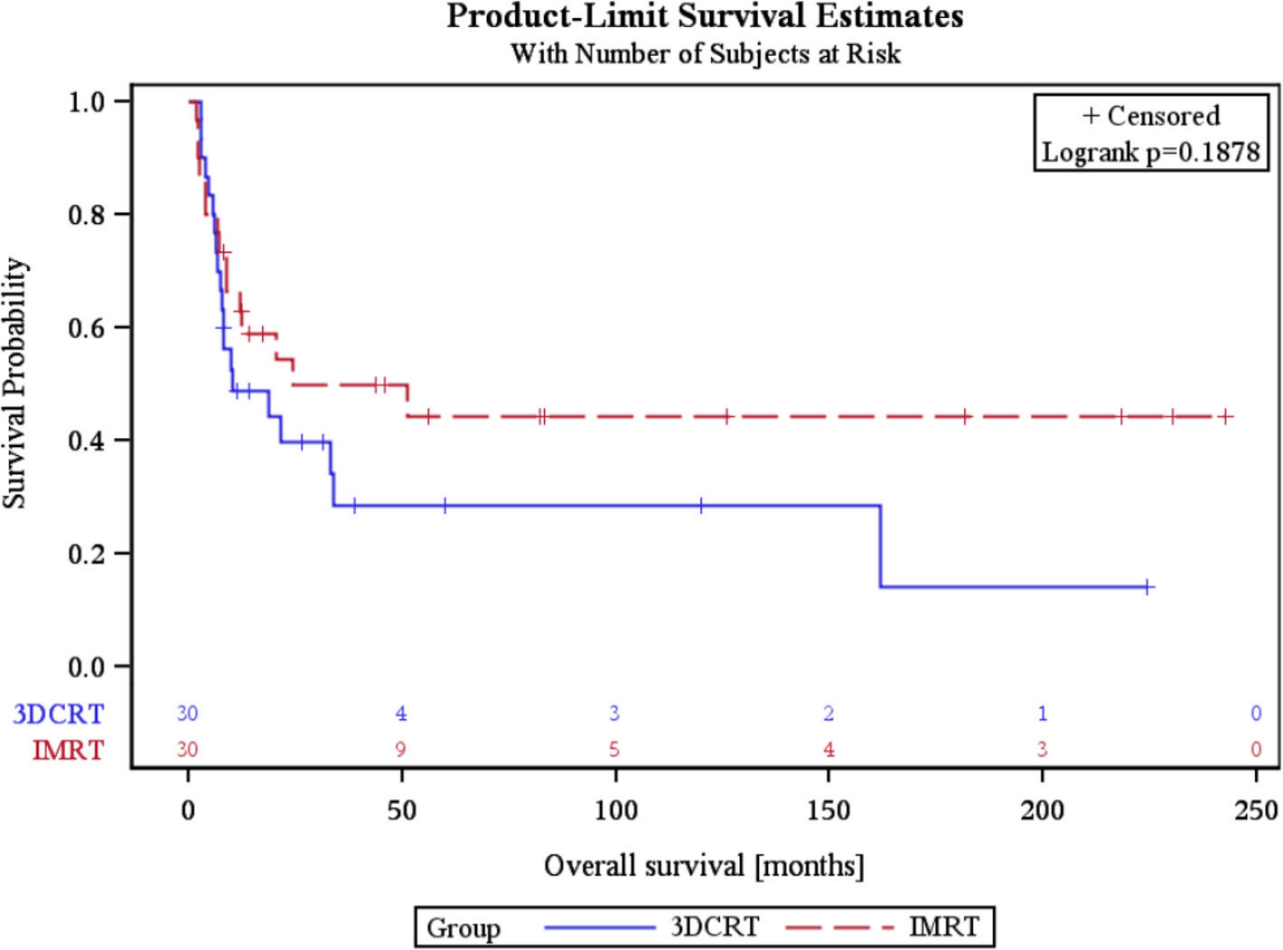
Overall survival of both arms

Although all surviving patients completed all assessments, not all patients survived by the three and six month time periods. Within the first 3 months, 10 patients (33.3%) in the IMRT group had died, along with 11 patients (36.7%) in the 3DCRT arm. Between 3 and 6 months, another 2 patients (10%) died from tumor progression in the IMRT cohort, along with a further 7 patients (36.8%) in the 3DCRT arm (Fig. 1). OS did not differ between groups (*p* = 0.187) (Fig. 2). The mean follow-up was 6.3 months (IQR 2.5–9.3) for both groups.

As compared to baseline, bone density became significantly higher at 3 and 6 months following IMRT by a median percentage of 24.8% and 33.8% (*p* < 0.01 for 3 months and *p* = 0.048 for 6 months), respectively (Table 2). These figures in the 3DCRT cohort were 18.5% and 48.4% (p < 0.01 for both), respectively. There were no statistical differences in bone density between IMRT and 3DCRT at 3 (*p* = 0.723) or 6 months (*p* = 0.341).

**Table 2:** Bone density of both groups in metastatic bone before RT (baseline), as well as 3 and 6 months after RT

	IMRT group			Within group	3DCRT group			Within group	Differences between groups		
	*n*	Median	SD	*p*-value	*n*	Median	SD	*p*-value	HL	95% CI	*p*-value
All metastases											
HU Baseline	30	258.5	183.3		30	195.0	125.4		62	5.0–126.0	0.037
HU T2	20	419.3	232.7		19	300.0	165.7		−59.5	−194.0-59.0	0.232
HU T3	18	416.8	277.7		12	454.0	185.4		61.5	− 146.0-229	0.641
3 months											
HU T0-T2	20	90.5	134.2	< 0.01	19	35.0	87.1	< 0.01	−25.0	− 86.0-42.0	0.407
HU T0-T2 (%)	20	24.8	51.0	< 0.01	19	18.5	38.7	< 0.01	−4.5	−25.5-21.9	0.723
6 months											
HU T0-T3	18	124.0	166.0	0.023	12	132.0	157.7	< 0.01	59.0	−73.0-193.0	0.330
HU T0-T3 (%)	18	33.8	61.6	0.048	12	48.4	70.7	< 0.01	34.0	−20.3-91.1	0.341

The results were presented by absolute and relative values (%) of HU within and between groups as median (Hodges–Lehmann estimate) and IQR

Subgroup evaluation of solely osteolytic lesions at 3 and 6 months showed no significant differences between groups (*p* = 0.489 and *p* = 0.377 respectively) (Table 3). There were no differences between bone density changes in osteoblastic metastases in the IMRT and 3DCRT groups at 3 or 6 months (*p* = 1.000 for both) (Table 3). Subgroup evaluation of mixed lesions showed a significant difference (*p* = 0.037) at 6 months but not at 3 months (*p* = 0.256) (Table 3).

**Table 3:** The subgroup analysis of the bone density (HU = Hounsfield units) in metastatic bone before RT (baseline), as well as 3 and 6 months after RT

	IMRT group			3DCRT group			Differences between groups		
	*n*	Median	SD	*n*	Median	SD	HL	95% CI	*p*-value
Mixed									
HU Baseline	17	242.0	100.1	10	230.5	77.3	−41.5	− 128.0-40.0	0.238
HU T2	11	360.0	153.1	5	418.0	89.4	58.0	− 114-204.0	0.450
HU T3	9	355.0	174.1	4	559.5	132.0	190.5	−61.0-416.0	0.190
3 months									
HU T0-T2	11	102.0	92.2	5	164.0	68.4	59.0	−43.0-174.0	0.213
HU T0-T2 (%)	11	38.8	41.2	5	60.8	48.5	25.7	−32.5-89.5	0.256
6 months									
HU T0-T3	9	176.0	123.1	4	301.0	89.4	161.5	73.0–341.0	0.025
HU T0-T3 (%)	9	55.0	61.1	4	145.9	42.2	80.7	8.7–155.1	0.037
Osteolytic									
HU Baseline	6	178.5	74.3	14	156.5	60.9	20.0	−60.0 − 82.0	0.536
HU T2	4	269.5	198.4	8	181.5	70.8	84.0	-82.0-387.0	0.270
HU T3	4	153.0	127.7	3	295.0	82.8	126.5	− 184.0-277.0	0.377
3 months									
HU T0-T2	4	35.5	171.2	8	16.0	38.0	19.5	−53.0-364.0	0.489
HU T0-T2 (%)	4	20.3	83.11	8	9.3	21.2	12.2	−27.5-178.4	0.489
6 months									
HU T0-T3	4	10.0	95.3	3	64.0	89.1	110.0	−60.0-315.0	0.212
HU T0-T3 (%)	4	−0.8	55.4	3	27.7	55.0	46.9	−61.2-174.2	0.377
Osteoblastic									
HU Baseline	7	419.0	247.6	6	426.0	159.8	−94.5	− 415.0-172.0	0.520
HU T2	5	574.0	327.0	6	481.5	174.4	145.5	− 284.0-604.0	0.411
HU T3	5	700.0	374.8	5	475.0	196.4	255.0	− 367.0-711.0	0.296
3 months									
HU T0-T2	5	52.0	188.3	6	13.0	103.3	45.0	− 311.0-155.0	0.647
HU T0-T2 (%)	5	15.4	35.8	6	3.1	27.5	3.0	−57.0-30.8	1.000
6 months									
HU T0-T3	5	130.0	241.7	5	57.0	102.9	42.0	− 408.0-224.0	0.676
HU T0-T3 (%)	5	15.4	47.2	5	13.6	27.3	1.7	−77.9-53.4	1.000

The results were presented by absolute and relative values (%) of HU within and between groups as median (Hodges–Lehmann estimate) and IQR. Abbreviations: *HU* Hounsfield units, *IQR* interquartile range, *T0* baseline, *T2* 3 months, *T3* 6 months, *T0–T2* difference in baseline minus 3 months, *T0-T3* difference in baseline minus 6 months

Bone density in unaffected vertebrae did not show substantial changes within groups at 3 and 6 months following RT (IMRT: *p* = 0.623 and *p* = 0.167, 3DCRT: *p* = 0.934 and *p* = 0.147). There were also no significant differences between the IMRT and 3DCRT arms at 3 (*p* = 0.574) or 6 months (*p* = 0.949).

Preexisting pathological fractures existed in 3.3% patients in the IMRT arm vs. 13.3% in the 3DCRT group (*p* = 0.161) (Table 4). By 3 and 6 months, these numbers rose to 15.0% vs. 10.5% (*p* = 0.676) and 16.7% vs. 16.7% (p = 1.000), respectively. No pathological fractures in either group required salvage surgical intervention.

**Table 4:** Results of pathological fractures of both groups

	IMRT group			3DCRT group			Differences between groups		
	*n*	*n*/(%)		*n*	*n*/(%)		*p*-value		
		No	Yes		No	Yes			
Baseline	30	29 (96.7%)	1 (3.3%)	30	26 (86.7%)	4 (13.3%)	0.161		
3 months	20	17 (85.0%)	3 (15%)	19	17 (89.5%)	2 (10.5%)	0.676		
6 months	18	15 (83.3%)	3 (16.7%)	12	10 (83.3%)	2 (16.7%)	1.000		

Abbreviations: n = alive participants; n/% = total number of pathological fractures in absolute and percentage terms

Pain assessment, using VAS scoring, was similar between cohorts at baseline (*p* = 0.882) and immediately following RT (*p* = 0.075). Although the IMRT arm showed improved pain response at 3 months (p = 0.037), this was not observed at 6 months (*p* = 0.430). There were also no differences in neuropathic pain at 3 (*p* = 0.946) or 6 (*p* = 0.661) months. Using Chow criteria, pain response was statistically similar at both 3 (*p* = 0.395) and 6 (*p* = 0.732) months (Table 5). At 3 months, 70.0% of patients that underwent IMRT were categorized as responders, as compared to 47.4% in the 3DCRT arm (*p* = 0.151); these numbers at 6 months were 70.8% and 58.3%, respectively (*p* = 0.494).

**Table 5:** Response according to Brief Pain Inventory score at 3 and 6 months in the per-protocol cohort

	IMRT group *n* = 20			3DCRT group *n* = 19		
After 3 months	n	%		n	%	*p*-value
CR	10	50		5	26,3	0.395
PR	4	20		4	20,1	
PP	1	5		3	15,8	
IP	5	25		7	36,8	
Responders	14	70		9	47,4	0.151
Non-responders	6	30		10	52,6	
	IMRT group *n* = 17			3DCRT group *n* = 12		
After 6 months						
CR	7	41,2		3	25	0.732
PR	5	29,4		4	33,3	
PP	2	11,8		3	25	
IP	3	17,7		2	16,7	
Responders	12	70,8		7	58,3	0.494
Non-responders	5	29,4		5	41,7	

Abbreviations: *CR* complete response, *PR* partial response, *PP* pain progression, *IP* intermediate pain

**Fig. 3:**
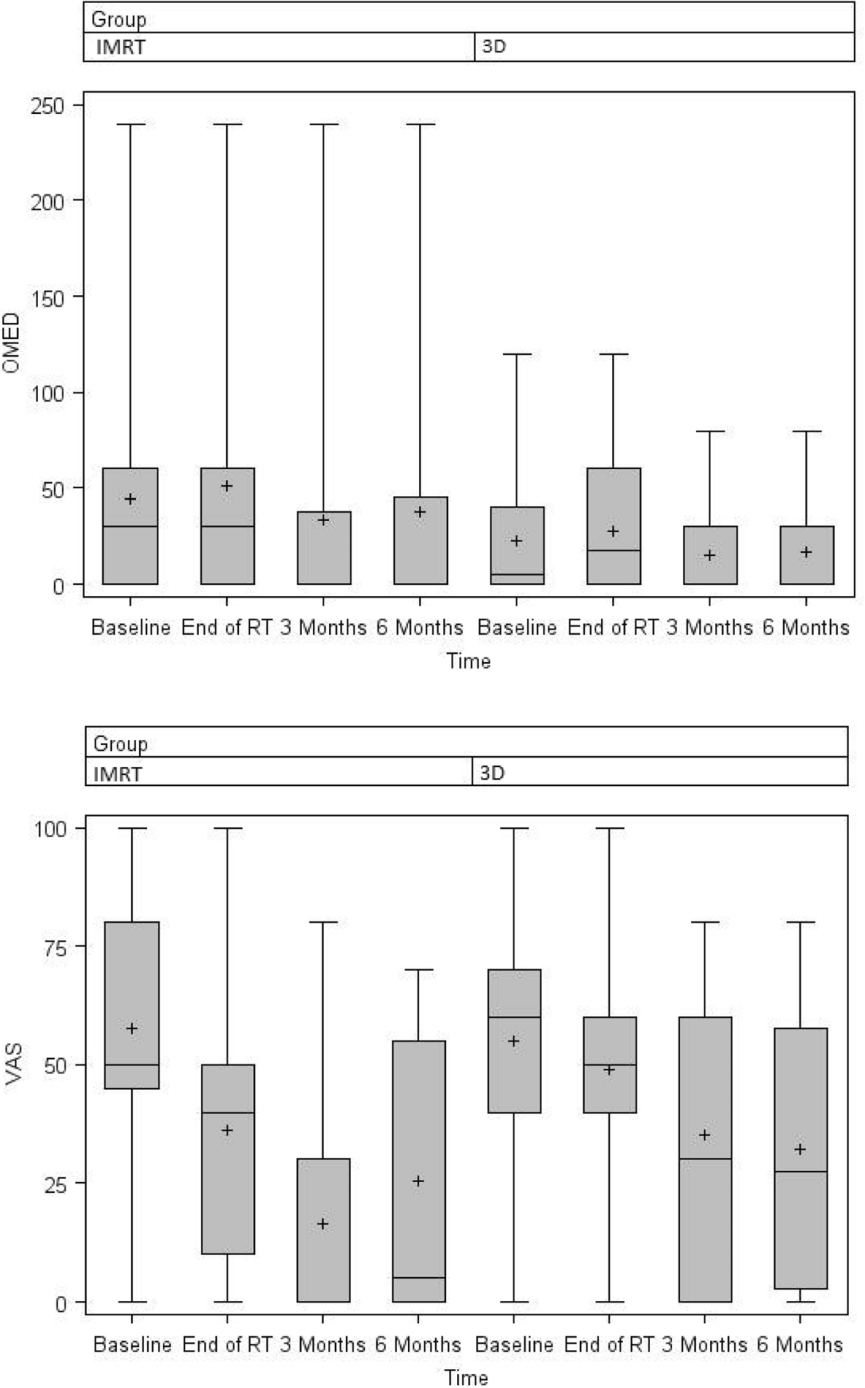
OMED and VAS of both groups at measured points

There were no differences in the pattern of recorded OMED consumption between groups at both 3 and 6 months after RT (Fig. 3).

## Discussion

This prespecified secondary evaluation of a prospective randomized trial is the first to investigate the impact of image-guided IMRT on bone density as compared to 3DCRT. No differential effects on bone density or other secondary endpoints were expected between IMRT and 3DCRT techniques at the time of study creation. The significant rises in bone density after 3 and 6 months, along with pathologic fracture rates and pain response, were similar in both cohorts.

Despite rightful concerns regarding its cost-effectiveness for palliative vertebral irradiation, IMRT is an attractive option in part owing to the ability to perform simultaneous integrated boosting (SIB). This refers to allowing multiple target volumes to receive different doses per fraction, while maintaining the same total number of fractions. Although no patient in this trial received SIB, this topic will be better understood following maturation of the IRON-2 trial, which consists of four arms: 20 Gy in 5 fractions (with or without SIB to 30 Gy in 5 fractions) and 30 Gy in 10 fractions (with or without SIB to 40 Gy in 10 fractions) [8]. Evaluating bone density in such instances will be essential to evaluate whether higher fractional doses are safe from the bone density standpoint as well.

From these data, it was noteworthy that baseline bone density was higher in the IMRT arm (*p* = 0.037). Although numerically higher at 3 months as well (median 419 versus 300), this did not reach statistical significance (*p* = 0.232), likely owing to the lower sample sizes available at 3 months. Nevertheless, 6 month values were numerically comparable. Moreover, the relative magnitude of bone density change at 3 and 6 months was also numerically and statistically comparable between both groups. This implies that components of bone density changes specifically related to RT are likely similar between both cohorts.

What is less clear are the findings related to mixed osteolytic-osteoblastic lesions at 6 months. The three-month values were not significantly different between cohorts, nor were those for purely osteolytic or osteoblastic metastases. As such, these analyses with clearly small sample sizes may not provide robust conclusions in this subgroup of patients. Additionally, only a few previous studies included mixed or osteoblastic metastases. In contrast to our results, Eggermont et al. did not observe any bone density changes in mixed proximal femur lesions after 4 and 10 weeks [9]. This could possibly result from an earlier date of collection and lower doses (prescription up to 20 or 24 Gy). In line with these results, less remineralization in the extremities was reported by Rieden and coauthors [10].

Wachenfeld et al. reported an increase in CT density in osteolytic metastases to approximately 150% of the initial value at 3 months after multi-fraction irradiation [11]. Koswig and Budach showed improvement of bone density in osteolytic metastases by 173% at 6 months after multi-fraction irradiation [12]. In this trial, however, the differences were 20.3% and 9.3% in the IMRT and 3DCRT arms, respectively, at 3 months; 6-month values were − 0.8% and 27.7%, respectively. There are several causes of these discrepancies, including the specific patient population, histology, size of metastases and several other factors.

The improved pain response based on VAS in the IMRT group (*p* = 0.037) at 3 months could have been from a greater use of hormonal therapy in metastatic prostate carcinoma. That being said, potential imbalances in anti-osteoresorptive therapies are an unlikely cause of the findings herein, as densities of unaffected vertebrae yielded no differences between groups. Rief et al. studied the impact of resistance training concomitantly with conventional multi-fraction 3DCRT on bone density in a randomized controlled study and found no significant differences in the uninvolved spine [13]. Therefore, it has been suggested that bisphosphonates may not exert decisive effects in this circumstance.

Despite the randomized design and standardized evaluation of bone density and recording of all pathological structures, several limitations must be noted. In addition to the lower sample size and shorter follow-up, robust conclusions based on statistical comparisons cannot be made, as partially discussed above. Second, a possible methodological weakness in our study was the lack of a control group. Third, many patients did not receive concurrent radiotherapy and chemotherapy, so the relationship of systemic therapy on bone density changes cannot be entirely excluded [14–18]. Fourth, some participants received immunotherapy and prior or subsequent radiotherapy to other distant metastases. The abscopal effect in this setting has been sporadically described but not sufficiently clarified [19, 20], but our study did not investigate this causality. Fifth, few studies can entirely account for other factors influencing bone density such as diet, particular medications, or vitamin supplementation. There may also be heterogeneity in this population given the specific location of vertebral metastases (e.g. vertebral body versus lamina/pedicle) as well as degree of soft tissue extension. Although these may limit applicability to other studies, larger randomized data are recommended to validate these results.

## Conclusions

This prespecified secondary evaluation of a prospective randomized trial is the first to investigate the impact of image-guided IMRT on bone density as compared to 3DCRT. The significant rises in bone density after 3 and 6 months, along with pain response and pathologic fracture rates, were similar in both cohorts. Future randomized investigations with larger sample sizes are recommended.

## Abbreviations

CR: Complete response
CT: Computed tomography
CTV: Clinical target volume
3DCRT: conventional 3D conformal radiotherapy
EBRT: external body radiotherapy
Gy: Gray
HU: Hounsfield unit
IMRT: Intensity-modulated radiotherapy
kV: kilo-voltage
MV: Megavoltage
SIB: simultaneous integrated boost
OAR: Organ at risk
OMED: oral equivalent morphine dose
OS: Overall survival
PP: Progression pain
PR: Partial response
PTV: Planning target volume
QoL: Quality of life
RT: Radiotherapy
SBRT: Stereotactic body radiation therapy
IP: Intermediate pain
SRS: Stereotactic radiosurgery
VAS: Visual analog scale
VCF: Vertebral compression fracture
VMAT: Volumetric modulated arc therapy
